# A Genome-Wide View of the Transcriptome Dynamics of Fresh-Cut Potato Tubers

**DOI:** 10.3390/genes14010181

**Published:** 2023-01-10

**Authors:** Li Wang, Wanxing Wang, Jianwei Shan, Chengchen Li, Haicui Suo, Jitao Liu, Kang An, Xiaobo Li, Xingyao Xiong

**Affiliations:** 1 Provincial Key Laboratory of Crops Genetic Improvement, Research Institute of Crops, Guangdong Academy of Agricultural Sciences, Guangzhou 510640, China; 2 Institute of Vegetables and Flowers, Chinese Academy of Agricultural Sciences, Beijing 100081, China; 3 Agricultural Genomics Institute at Shenzhen, Chinese Academy of Agricultural Sciences, Shenzhen 518000, China

**Keywords:** enzymatic browning, fresh-cut, potato tubers, transcriptome

## Abstract

Fresh fruits and vegetable products are easily perishable during postharvest handling due to enzymatic browning reactions. This phenomenon has contributed to a significant loss of food. To reveal the physiological changes in fresh-cut potato tubers at the molecular level, a transcriptome analysis of potato tubers after cutting was carried out. A total of 10,872, 10,449, and 11,880 differentially expressed genes (DEGs) were identified at 4 h, 12 h and 24 h after cutting, respectively. More than 87.5% of these DEGs were classified into the categories of biological process (BP) and molecular function (MF) based on Gene Ontology (GO) analysis. There was a difference in the response to cutting at different stages after the cutting of potato tubers. The genes related to the phenol and fatty biosynthesis pathways, which are responsible for enzymatic browning and wound healing in potato tubers, were significantly enriched at 0–24 h after cutting. Most genes related to the enzymatic browning of potato tubers were up-regulated in response to cut-wounding. Plant hormone biosynthesis, signal molecular biosynthesis and transduction-related genes, such as gibberelin (GA), cytokinin (CK), ethylene (ET), auxin (IAA), jasmonic acid (JA), salicylic (SA), and Respiratory burst oxidase (Rboh) significantly changed at the early stage after cutting. In addition, the transcription factors involved in the wound response were the most abundant at the early stage after cutting. The transcription factor with the greatest response to injury was MYB, followed by AP2-EREBP, C3H and WRKY. This study revealed the physiological changes at the molecular level of fresh-cut potato tubers after cutting. This information is needed for developing a better approach to enhancing the postharvest shelf life of fresh processed potato and the breeding of potato plants that are resistant to enzymatic browning.

## 1. Introduction

The enzymatic browning process is a natural phenomenon occurring in fruits and vegetables, which has become a challenge in the food industry sector. The occurrence of food browning reactions usually impairs the colour appearance of food and has markedly reduced the customer’s acceptance of the products, which causes heavy economic losses to both food producers and the food-processing industry, as this process mostly occurs during postharvest handling, transportation, storage and processing [1]. According to a report, enzymatic browning may be responsible for more than 50% of the foods that are susceptible to browning being wasted [2].

Enzymatic browning often occurs on the surface of wounded tissues during fruit and vegetable processing, which always leads to a decline in the appearance, flavor and nutritional quality of most fruit and vegetable products, with the exception of tea, and is therefore considered the second largest cause of quality loss [3,4]. Polyphenol oxidase (PPO) and peroxidase (POD) are the main enzymes that cause the enzymatic browning of fruits and vegetables [5,6]. PPO and POD can catalyze substrates such as phenols, anthocyanins and flavonoids to form quinones in the presence of oxygen and hydrogen peroxide, respectively [7,8,9]. The highly reactive quinones can then polymerize with themselves, proteins, amino acids or other macromolecular substances to produce black, brown or red pigments that accumulate on the surface of wounded tissues [10]. In general, the oxidizing enzyme is separated from its substrate. When fruit and vegetable tissue is damaged by some stress, such as cold, age, or mechanical injury, its cells compartmentalize loss; consequently, phenolic components are released and interact with oxidizing enzymes, leading to browning reactions in a short time. In addition, antioxidant capacity, such as that provided by the activities of Catalase (CAT), superoxide dismutase (SOD), glutathione peroxidase (GPX), and ascorbate peroxidase (APX), and the contents of reactive oxygen species (ROS), including hydrogen peroxide (H_2_O_2_) and hydroxyl radicals (OH•) and superoxide anions (O2•−), have been suggested to affect browning development in many reports [11,12,13,14,15]. SOD and CAT are important and indispensable antioxidant enzymes in many defense strategies in plants [16]. SOD catalyzes the dismutation of the superoxide anion (O2•–) to H_2_O_2_, which can be decomposed by CAT, thus reducing the ROS content.

Potato (*Solanum tuberosum* L.) is widely grown in more than 150 countries and regions worldwide, and has become the third largest food crop consumed after rice and wheat (http://faostar.fao.org (accessed on 28 December 2021)). It is the only tuber crop that can be used as both a vegetable and staple food, and will be instrumental in achieving global food security due to its comprehensive nutritional value, wide growth adaptability and high yield [17]. Cut-wounding is inevitable during fresh potato processing. However, a series of physiological and biochemical reactions, especially enzymatic browning, occur during the processing of fresh-cut potatoes, which leads to a serious loss of their edible and nutritional value [14]. Therefore, to reveal the series of physiological changes in potato tubers after cutting at the transcriptome level, transcriptome sequencing was performed on potato tubers at different times after cutting in this study. This information is needed to develop better approaches for enhancing the postharvest shelf life of fresh-processed potato and its storage after harvesting, as well as the breeding of potato plants resistant to enzymatic browning. As Cisneros-Zevallos and Jacobo-Velázquez indicated (2020) [18], studies on the molecular, physiological, and biochemical basis of controlled abiotic stresses will enable the improvement and development of novel technologies in both pre- and postharvest applications.

## 2. Materials and Methods

### 2.1. Plant Materials and Determination of Browning Degree of Potato Tubers

The tetraploid potato cultivar Xingjia 2 (X2) was used in this study and was grown in the Baiyun District Experimental Field, Guangdong Province, China. Approximately 30 d after harvest, tubers free from apparent diseases and mechanical injury were selected and cut into two transverse halves, and then kept in trays with about 90 (±2)% relative humidity and 25 °C temperature. The enzymatic browning of potato tubers was determined using a colorimeter (NS810, 3nh, Shenzhen, China) by referring to the method of Wang et al. (2020) [19]. The ΔL*, Δa*, Δb*, and ΔE* were recorded at 0 h, 1 h, 2 h, 4 h, 6 h, 8 h, 12 h and 24 h after cutting.

At different time points (0, 4, 12, 24 h) after cutting, approximately 5 mm thick blocks of tuber tissues were radially cut from the epidermis to the pith, flash frozen in liquid nitrogen and stored at −80 °C for RNA extraction. Samples collected at 0 h after cutting were used as controls. Three biological replicates were performed for each sample. Each biological replicate is a mix of samples from three tubers.

### 2.2. RNA Extraction, Library Construction and Sequencing

The total RNA of potato tubers was extracted from each sample using a TiangenRNA extraction kit (Tiangen, Beijing, China) according to the manufacturer’s protocol. RNA degradation and contamination were monitored on 1% agarose gels. The purity, concentration, and integrity of RNA samples were measured as described by Zhao et al. (2016) [20]. For library construction and sequencing, refer to the method described by Fu et al. (2021) [21]. All raw data were submitted to the Gene Expression Omnibus (GEO) (No. GSE178602).

### 2.3. De Novo Assembly, DEG Analysis and Annotation

Clean reads were obtained by removing reads containing adapters, reads containing poly-N sequences and low-quality reads from the raw data, and were then used for de novo assembly with Trinity (http://trinityrnaseq.sourceforge.net/ (accessed on 14 July 2014)) [22]. At the same time, the Q20, Q30 and GC content of the clean data were calculated. All downstream analyses were based on high-quality clean data. Clean reads were then mapped to the potato reference genome (SolTub_3.0) using HISAT software [23,24]. Feature Counts v1.5.0-p3 was used to count the read numbers mapped to each gene [25]. The FPKM of each gene was then calculated based on the length of the gene and the number of reads mapped to the gene. The R package DESeq was used to identify DEGs between the samples based on a |Log2fold change| ≥ 1 and *p*-value ≤ 0.01 [26].

To annotate the DEGs, all of the assembled transcripts were searched against the publicly available Kyoto Encyclopedia of Genes and Genomes (KEGG) and GO databases using a corrected *p*-value ≤ 0.05 to obtain optimal functional annotation. The BLAST2GO (http://www.blast2go.com/b2ghome (accessed on 1 June 2021)) program was used to obtain the GO annotations of the unique assembled transcripts [27]. A metabolic pathway analysis was performed using KEGG (http://www.genome.jp/kegg/ (accessed on 22 September 2021)) [28].

### 2.4. Quantitative Real-Time PCR (qRT-PCR) Analysis

Total RNA was extracted from the samples using a Tiangen RNA extraction kit (Tiangen, Beijing, China) according to the manufacturer’s protocol. Approximately 2 μg of total RNA was reverse transcribed using a Tiangen trans kit (Tiangen, Beijing, China) following the manufacturer’s protocol and stored at −20 °C for qRT-PCR. A qRT-PCR analysis was performed using the Premix Ex TaqTM kit (Takara, Dalian, Japan) on the StepOne plus PCR platform (Applied Biosystems). The PCR program refers to our previous study [19]. *EF1α* was used as the internal control [29]. Relative changes in gene expression were assessed using the 2^−∆∆Ct^ method. Three biological replicates were performed for each treatment, and three technical replicates were performed for each sample. The sequences of the primers are listed in Supplementary Materials Table S1.

### 2.5. Determination of Enzyme Activity and H_2_O_2_ Content

Samples were collected from the perimedullary of potato tubers at different times (0 h, 4 h, 12 h, 24 h) after cutting for the assay of PPO, POD, Phenylalanine ammonia-lyase (PAL), CAT, SOD activity and H_2_O_2_ content. PPO, POD, PAL, CAT, SOD activity and H_2_O_2_ content was calorimetrically measured using an assay kit (Product code: BC0190, BC0090, BC0210, BC4780, BC0170, BC3590, respectively; Beijing Solarbio Science & Technology Co. Ltd., Beijing, China) and calculated according to the manufacturer’s instructions.

### 2.6. Statistical Analysis

All experiments were performed at least in triplicate, and the data were expressed as mean ± standard error of mean (SE). The statistical analysis was performed by one-way analysis of variance using BMC SPSS version 25 (SPSS Inc, Chicago, IL, USA). The significant differences at *p* < 0.05 were obtained using Duncan’s multiple range test.

## 3. Results

### 3.1. Browning Characteristic of Potato Tubers at Different Times after Cutting

**Figure 1:**
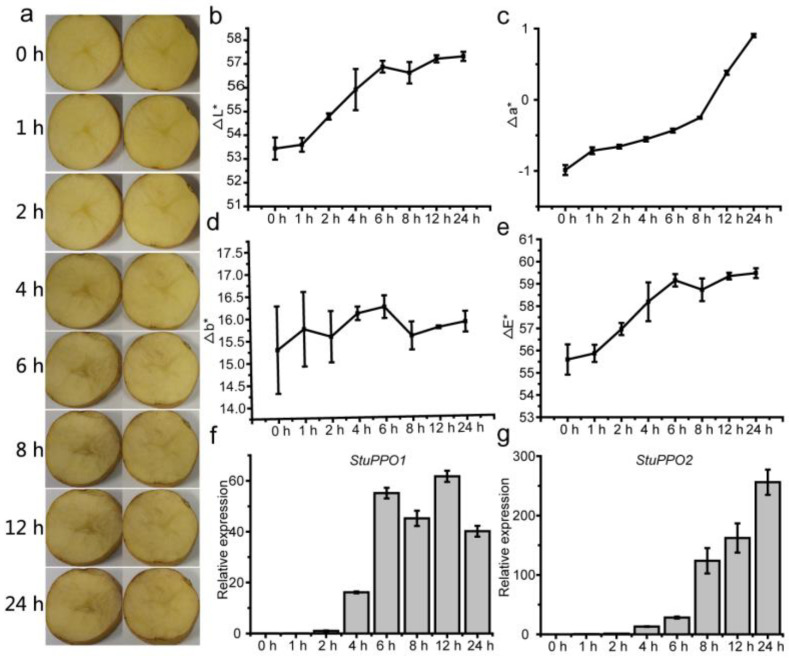
The changes of phenotype and related indexes of potato tuber during enzymatic browning. (**a**), Browning characteristic of potato tubers at different times; (**b**–**e**), the changes of ΔL*, Δa*, Δb*, and ΔE* of potato tubers during enzymatic browning, respectively; (**f**,**g**), The expression of PPO genes at different times in potato tubers after cutting. Values are means ± SE (n = 3).

The tubers of X2 were cut transversely and observed enzymatic browning at different times. Obvious enzymatic browning began at 4 h after cutting and deepened over time (Figure 1a). Except Δb*, the values of ΔL*, Δa* and ΔE* increased over time (Figure 1b–e). Furthermore, we detected the expression of PPO genes (*StuPPO1*, *StuPPO2*) of potato reported previously [30], and the results showed that the expression of both SuoPPO genes increased significantly at 4 h after cutting, and then were further upregulated over time (Figure 1f,g).

Based on the above, the total RNA of potato tuber samples at 0, 4, 12, and 24 h after cutting was extracted, and an RNA-Seq was performed.

### 3.2. Transcriptome Data Generation and Processing

A total of 566.23 million raw reads were generated (Supplementary Materials Table S2). After removing the low-quality reads and adaptor sequences, a total of 557.68 million clean reads were detected across all samples of the X2 cultivar, which yielded 83.66 Gb of nucleotide data. The GC contents were 42.29–43.50%. The percentage of Q20 and Q30 were 96.30–97.55% and 90.54–93.23%, respectively. We mapped the clean reads to the potato reference genome, and 84.74–87.66% of the clean reads successfully mapped to the reference genome (Supplementary Materials Table S3). Most of the total reads were uniquely mapped to the potato genome, whereas a small proportion (approximately 2.61–4.04% of the total mapped reads) was mapped to multiple locations in the potato genome. The Pearson correlation coefficient between the three replications was more than 0.986 (Supplementary Materials Figure S1), indicating that the gene expression in the three replications of each sample was homogeneous. A principal component analysis (PCA) was also performed to determine the outliers of RNA-seq data, and showed the good repeatability between biological repeats (Supplementary Materials Figure S2). Furthermore, several DEGs were randomly selected to verify their expression via qRT-PCR, the results of which were in accordance with the RNA-seq results (Supplementary Materials Figure S3). Overall, these results suggest that our transcriptome analysis using RNA-Seq was reliable, and the sequencing output and quality were sufficient to warrant further analysis.

### 3.3. DEG Analysis of Potato Tubers after Cutting

**Figure 2:**
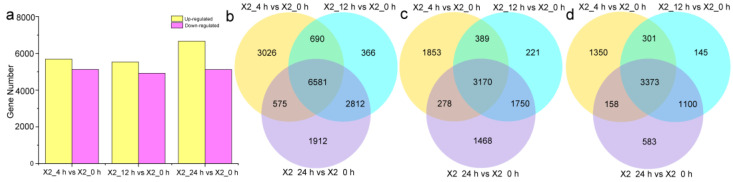
Statistical analysis of all identified DEGs in response to cut-wounding at different times after cutting. (**a**), Statistical chart of DEGs of transcriptomes in response to cut-wounding at 4 h, 12 h, and 24 h after cutting; (**b**–**d**), Venn diagram of total DEGs (**a**), up-regulated DEGs (**b**) and down-regulated DEGs at different times after cutting.

A differential gene expression analysis based on |log2FC| ≥ 1 and a *p*-value ≤ 0.01 identified 10,872 (5690 up-regulated and 5182 down-regulated), 10,449 (5530 up-regulated and 4919 down-regulated), and 11,880 (6666 up-regulated and 5214 down-regulated) DEGs at 4, 12, and 24 h compared to 0 h after cutting, respectively (Figure 2a). A complete list of DEGs is provided in the supplementary datasets (Supplementary Materials Table S4). In general, the number of up-regulated DEGs was significantly higher than that of the down-regulated DEGs at three time points after cutting. After cutting, the number of up-regulated genes increased gradually and reached a maximum at 24 h, while the number of down-regulated genes decreased gradually, reached a minimum at 12 h, and then increased, indicating a difference in different response stages after wounding. A Venn diagram analysis showed that 6581 DEGs (3170 up-regulated and 3373 down-regulated) were shared across the three time points after cutting (Figure 2b–d). This result indicated that 6581 DEGs were involved in the continuous response 0–24 h after cutting. There were 3026, 366, and 1912 specific DEGs at 4 h, 12 h and 24 h after cutting, respectively (Figure 2b). The most DEGs were found at 4 h after cutting, followed by 24 h, and the least were found at 12 h after cutting. Specific DEGs at the three time points represent specific changes at different stages after cutting.

### 3.4. GO and KEGG Enrichment of DEGs in Response to Cut-Wounding

**Figure 3:**
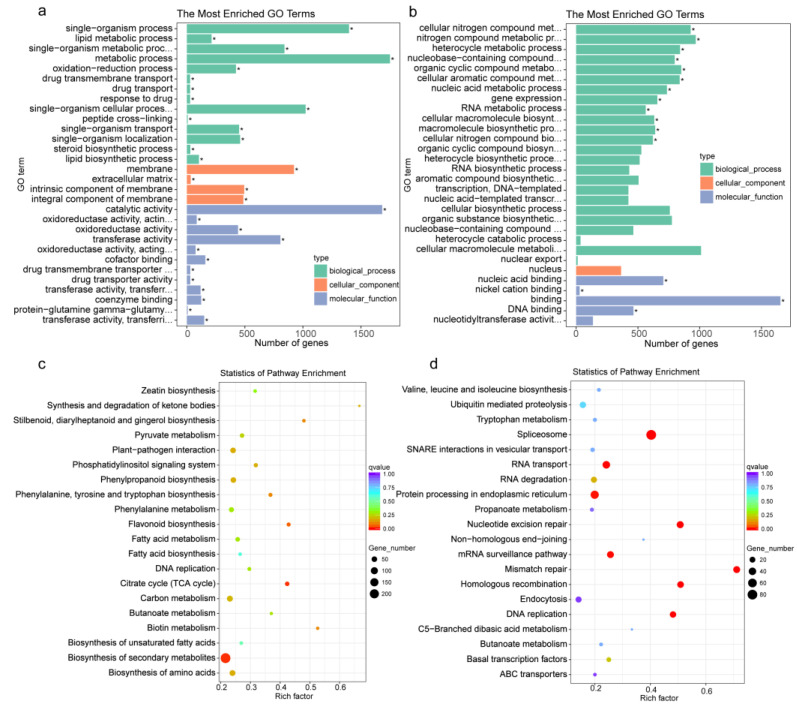
GO and KEGG analysis of common DEGs at different times in response to cut-wounding. (**a**,**b**), GO terms of the functional categorization of up- and down-regulated DEGs of all common DEGs at different time in response to cut-wounding, respectively; Significantly enriched GO terms are indicated by * for a *p*-value less than and equal to 0.05; (**c**,**d**), KEGG classification of the functional categorization of up- and down-regulated DEGs of all common DEGs at different times in response to cut-wounding, respectively.

We conducted a GO analysis on 6581 shared DEGs. The results showed that 3170 up-regulated DEGs were enriched across the three categories (Figure 3a and Supplementary Materials Table S5). More than 87.5% of these DEGs were classified into the categories biological process (BP) and molecular function (MF) based on Gene Ontology (GO) analysis. In BP, metabolic process was the most dominant term, followed by single-organism process, single-organism cellular process, single-organism metabolic process, single-organism localization, single-organism transport, oxidation-reduction process, lipid metabolic process, lipid biosynthetic process, steroid biosynthetic process, drug transmembrane transport, drug transport, and response to drug. In MF, catalytic activity; oxidoreductase activity, acting on CH-OH group of donors; oxidoreductase activity; transferase activity; oxidoreductase activity, acting on the CH-OH group of donors; NAD or NADP as acceptor; cofactor binding; drug transmembrane transporter activity; drug transporter activity; transferase activity; transferring acyl groups; coenzyme binding, protein-glutamine γ-glutamyltransferase activity; transferase activity, and transferring glycosyl groups were the most enriched. In CC, membrane-related functional terms were the most enriched.

An analysis of the 3373 shared down-regulated DEGs was also performed (Figure 3b and Supplementary Materials Table S5). In BP, cellular nitrogen compound metabolic process, nitrogen compound metabolic process, heterocycle metabolic process, nucleobase-containing compound metabolic process, organic cyclic compound metabolic process, cellular aromatic compound metabolic process, nucleic acid metabolic process, gene expression, RNA metabolic process, cellular macromolecule biosynthetic process, macromolecule biosynthetic process, cellular nitrogen compound biosynthetic process, organic cyclic compound biosynthetic process, heterocycle biosynthetic process, RNA biosynthetic process, aromatic compound biosynthetic process, transcription, DNA template, nucleic acid template transcription, cellular biosynthetic process, organic substance biosynthetic process, nucleobase-containing compound biosynthetic process, heterocycle catabolic process, cellular macromolecule metabolic process, and nuclear export were the most enriched. In MF, nucleic acid binding, nickel cation binding, binding, DNA binding, and nucleotidyltransferase activity were enriched. In CC, nucleus was the most enriched. These results indicated that the biological functions described above might be activated under wounding stress in potato tubers.

To better understand the biological functions of our DEGs in their respective biochemical pathways, we also performed a KEGG analysis for 6581 shared DEGs. The results showed that the 3170 up-regulated DEGs were enriched in 116 biological pathways (Figure 2c and Supplementary Materials Table S5). Among these significantly enriched pathways, 235 DEGs were mostly enriched in the biosynthesis of secondary metabolites, and 25 DEGs were enriched in the citrate cycle (TCA cycle). Phenylpropanoid biosynthesis, stilbenoid, diarylheptanoid and gingerol biosynthesis, and flavonoid biosynthesis, which produce defensive secondary metabolites, were also enriched. Furthermore, amino acid-related pathways, such as phenylalanine metabolism, phenylalanine, tyrosine and tryptophan biosynthesis and the biosynthesis of amino acids, were enriched for 38, 18 and 29 DEGs, respectively. In addition, fatty acid-related pathways, such as fatty acid metabolism, fatty acid biosynthesis, butanoate metabolism and synthesis and degradation of ketone bodies, were also enriched. The 3373 down-regulated DEGs were enriched in 109 biological pathways (Figure 3d and Supplementary Materials Table S5), and significantly enriched in the spliceosome, mismatch repair, nucleotide excision repair, homologous recombination, DNA replication, RNA transport, mRNA surveillance, and protein processing pathways in the endoplasmic reticulum.

Furthermore, we performed a GO analysis for the specific DEGs at the three time points after cutting (Supplementary Materials Table S6 and Figure S4). At 4 h, 3026 specific DEGs were enriched in one or more GO terms. Among them, 906 DEGs were enriched in the functional term ion binding; the specific DEGs at 12 h were mainly enriched in DNA metabolic process, DNA binding, and nucleic acid binding; at 24 h, 1912 specific DEGs were mostly enriched in single-organism metabolic process, metabolic process, and catalytic activity. These results implied that there were some differences in the changes in potato tubers at different stages after cutting.

### 3.5. Antioxidant Enzymes and Enzymatic Browning-Related Genes in Response to Cut-Wounding

**Figure 4:**
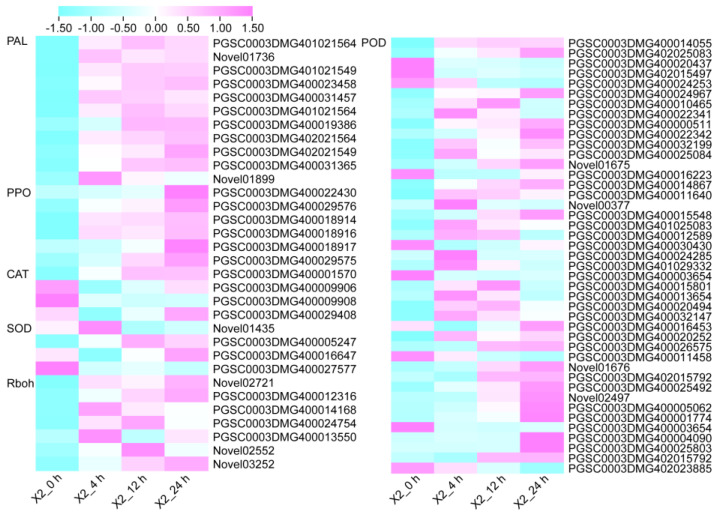
The expression heatmap of antioxidant enzymes−related genes based on the values of FPKM. PAL, Phenylalanine ammonialyase; PPO, polyphenol oxidase; CAT, Catalase; SOD, superoxide dismutase; POD, peroxidase.

Phenylalanine ammonialyase (PAL) is the first and rate-limiting enzyme of the phenylpropanoid biosynthesis pathway, and it is also the key enzyme in the production of phenolic compounds. In this study, we detected 11 PAL genes, all of which were up-regulated in response to cut-wounding and reached their highest levels at 12 h or 24 h. PPO is the key enzyme in the enzymatic browning of potato tubers. Similar to PAL genes, six PPO genes detected were also up-regulated in response to cut-wounding and reached their highest expression at 24 h after cutting, with a log2FC of 3.099–7.305. CAT, SOD, and POD can remove ROS from plants under stress. In this study, we detected four CAT genes, four SOD genes and 43 POD genes. Among the four CAT genes, one gene was up-regulated after cutting; the expression levels of two genes were the lowest at 4 h after injury and then increased. Among the four SOD genes, except for PGSC0003DMG400005247, the others were down-regulated. Among the 43 POD genes, 32 (74.4%) were up-regulated, and the rest were down-regulated (Figure 4 and Table S7). Rboh is the key enzyme in ROS production. Seven Rboh genes were detected, all of which showed up-regulated expression. Among them, the Log2FC of PGSC0003DMG400024754 is greater than 4.9.

### 3.6. DEGs with a |Log2FC| ≥ 6

We focused on the unigenes that were strongly induced by cut-wounding. The 898 DEGs with a |Log2FC| ≥ 6 were detected (Table 1 and Table S8). Of these, 128 genes were down-regulated and 770 genes were up-regulated, accounting for 85.7% of the total. KEGG analysis indicated that the functions of these DEGs were divided into four related categories, including plant defense, wound healing, DNA synthesis and degradation, and signal synthesis and transmission. The phenylpropanoid biosynthesis pathway, which contributes to the biosynthesis of phenol compounds and lignin, contained 15 DEGs with a |log2FC| ≥ 6, including one gene encoding trans-cinnamate 4-monooxygenase, four genes encoding cinnamyl-alcohol dehydrogenase, one gene encoding β-glucosidase, one gene encoding coniferyl-alcohol glucosyltransferase, and eight genes encoding peroxidase. In addition, the starch and sucrose metabolism and plant hormone signal transduction pathways contained 10 DEGs, respectively. Furthermore, some typical genes encoding defense-related proteins were also found, such as chitinase, which was up-regulated in response to cut-wounding and belonged to the amino sugar and nucleotide sugar metabolism pathway. Some key genes for plant hormone biosynthesis, including GA, CK, and ET, were also found, which belonged to the diterpenoid biosynthesis, zeatin biosynthesis, and cysteine and methionine metabolism pathways, respectively. It was also observed that the expression of genes related to the signal transduction of IAA (5), JA (2), and SA (1) was strongly altered in response to cut-wounding. As an example, the expression of a gene encoding one auxin-responsive GH3 gene family member, four genes encoding SAUR family proteins, and two genes encoding jasmonate ZIM domain-containing proteins was up-regulated after cutting.

### 3.7. Transcription Factor Genes Identified in Response to Cut-Wounding

**Figure 5:**
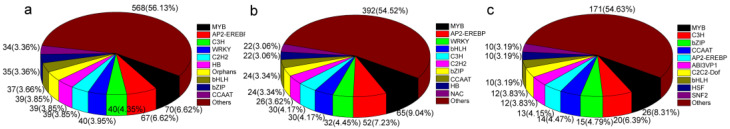
Classification and distribution of transcription factors in response to cut-wounding at different times. (**a**), (**b**), and (**c**), 4 h, 12 h, and 24 h after cutting, respectively.

Gene expression is often regulated at the transcriptional level. Therefore, the collection of DEGs was used to query PlantTFDB to identify transcription factors (TFs) that might have a vital role in the cut-wounding response. At 4 h, 12 h and 24 h after cutting, 1012, 719 and 313 TFs were found to be significantly responsive to cut-wounding, respectively (Figure 5 and Supplementary Materials Table S7). Over time, the number of transcription factors decreased, indicating that most transcription factors were mainly involved in the early wounding response. These TFs were assigned to 79 types. Among them, MYB was the most abundant at all three time points, followed by AP2-EREBP, C3H and WRKY, which indicated that these four types of TFs play important roles in the potato tuber wounding response. The number of up-regulated and down-regulated MYB transcription factors at 4 h and 12 h after cutting showed no significant difference. For example, at 4 h, 38 and 32 MYB transcription factors were up-regulated and down-regulated, respectively. At 12 h, 31 and 34 genes were up-regulated and down-regulated, respectively. However, all MYB transcription factor genes were down-regulated at 24 h. Unlike MYB, AP2-EREBP had far more up-regulated genes than down-regulated genes. For example, 54 and 13 of the AP2-EREBP transcription factor genes were up-regulated and down-regulated at 4 h, respectively, and 34 and 18 were up-regulated and down-regulated at 12 h, respectively. Similar to MYB, at 24 h, all of the AP2-EREBP TFs were down-regulated. The numbers of altered WRKY transcription factor genes at 4 h and 12 h were far greater than those at 24 h, which had only six WRKYs. In addition, 12 Aux/IAA transcription factors were involved in the wounding response. Based on the above analysis, transcription factors mainly respond in the early stage after cutting. Therefore, we further screened the TFs with a |log2FC| ≥ 6 at 4 h, and the results showed that these TFs included three MYB, two AP2-EREBP, two Tify, and one bHLH, LOB, HB, WRKY, E2F-DP, EIL, and MADS gene. These TFs may play an important role in the response to cut-wounding.

### 3.8. The Expression of Signal Molecular and Antioxidant Enzyme-Related DEGs

**Figure 6:**
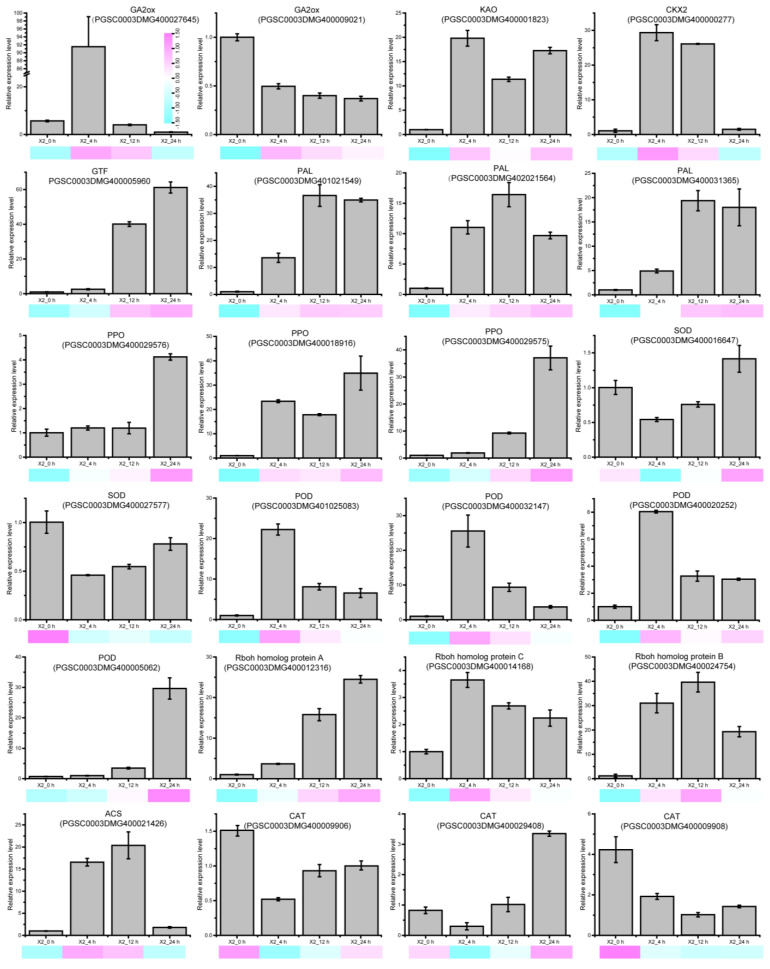
Expression profiles of signaling molecule biosynthesis, enzymatic browning related DEGs in potato tubers after cutting. Cyan to purple indicates the FPKM values based on RNA-seq. Data are mean values ± SD (n = 3). GA2ox, gibberellin 2-oxidase; KAO, Ent-kaurenoic acid oxidase; CKX2, cytokinin oxidase/dehydrogenase 2; GTF, glucosyltransferase; PAL, phenylalanine ammonia-lyase; PPO, polyphenol oxidase; POD, peroxidase; CAT, Catalase; SOD, Superoxide dismutase; Rboh, Respiratory burst oxidase homolog protein; ACS, 1-aminocyclopropane-1-carboxylate synthase 3.

The expression pattern of signal molecular and antioxidant enzyme-related DEGs were selected and examined (Figure 6). Representative genes included GA biosynthesis related genes, such as the Gibberellin 2-oxidase gene and Ent-kaurenoic acid oxidase gene; CK biosynthesis related genes, such as Cytokinin oxidase/dehydrogenase 2 and Glucosyltransferase; ET biosynthesis related gene encoding 1-aminocyclopropane-1-carboxylate synthase 3; the Rboh genes, which are the key enzyme in ROS production; antioxidant enzyme-related genes, including the genes encoding PPO, POD, CAT, SOD; and the genes encoding the key enzyme of phenolic biosynthesis PAL. A great majority of these genes exhibited a significant change in expression levels after cutting.

After cutting, one gene encoding Gibberellin 2-oxidase up-regulated at 4 h after cutting and then decreased; another gene down-regulated continuously after cutting. The expression of genes encoding Cytokinin oxidase/dehydrogenase 2 and Glucosyltransferase, which are related to CK biosynthesis, increased after cutting except the Cytokinin oxidase/dehydrogenase 2 gene decrease at 24 h. All of the PPO and PAL genes up-regulated in response to cut-wounding. The POD genes, except PGSC0003DMG400029576, have the highest transcript level at 4 h after cutting. The expression of Rboh genes up-regulated in response to cut-wounding. The three genes encoding CAT have a higher transcript level at 0 h, and down-regulated in response to cut-wounding and then up-regulated. Although the exact fold change of the DEGs at several data points varied between RNA-Seq and qRT-PCR, the differential expression trends detected by the two approaches were largely consistent. Collectively, RT-qPCR data indicated that the expression of these genes were significantly induced by cut-wounding.

### 3.9. Enzyme Activity and H_2_O_2_ Content of Potato at Different Time after Cutting

**Figure 7:**
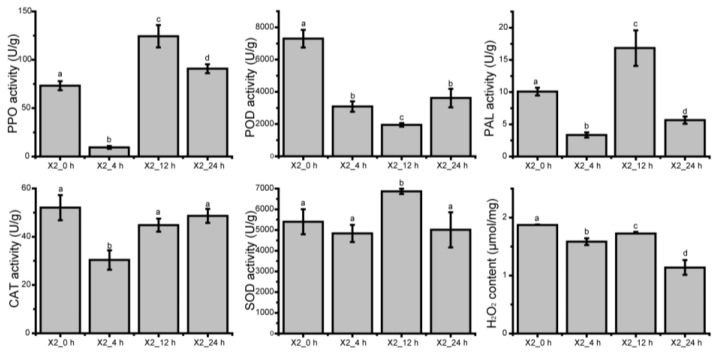
Enzyme activity and H_2_O_2_ content in potato tubers after cutting. Data are mean values ± SE for three independent replicates. Means denoted by different letters indicated a significant difference at *p* < 0.05 according to a Tukey’s test.

Furthermore, we detected the changes of PPO, POD, PAL, CAT and SOD activity and H_2_O_2_ content in potato tubers at different times after cutting (Figure 7). The results showed that the activity of PPO, POD, PAL and CAT decreased significantly within 4 h after cutting, and then increased, suggesting that new enzymes are synthesized as gene expression is up-regulated. The activity of SOD significantly increased at 12 h after cutting, and then decreased. The content of H_2_O_2_ showed a decreasing trend after cutting.

## 4. Discussion

Mechanical damage is common in fresh-cut fruits and vegetables during harvesting and processing. Plant tissue damage makes plants susceptible to microorganisms, which leads to the decay of plant tissues. However, plants produce secondary metabolites via a variety of physiological and molecular processes to form a protective layer at the wound site and adjacent tissues to prevent microorganism infection [31]. Furthermore, enzymatic browning on the surface of damaged tissues is a mechanism of plant self-protection, but it is harmful to the processing and preservation of agricultural products. In this study, we performed RNA sequencing in response to cut-wounding at different times based on X2 cultivars, a cultivar of potato widely cultivated in China, to understand the physiological changes after cutting at the transcriptome level.

### 4.1. DEGs Identified at Different Times after Cutting

A total of 33,201 DEGs were obtained from samples. The number of up-regulated DEGs was higher than that of down-regulated DEGs in response to cut-wounding, which is consistent with the results of Smith et al. (2004), who showed that the number of up-regulated genes after poplar wounding was greater than that of down-regulated genes [32]. There were 6581 DEGs shared across the three time points after cutting, indicating that these genes were part of a persistent response to cut-wounding for 24 h. Among them, there were 3170 up-regulated genes and 3373 down-regulated genes. Smith et al. (2004) [32] proposed that increased gene expression generates a suite of mechanisms that the plant uses to respond to a stimulus when the plant is wounded, while the repression of a subset of the genes suggests that resource reallocation takes place, thus remodeling growth and development in response to the stimuli. In our study, most of these up-regulated genes were enriched in the BP and MF categories. Among these, approximately 7.4% participated in the biosynthesis of the secondary metabolites pathway. Some of these genes were involved in lipid metabolism pathways, such as fatty acid metabolism, fatty acid biosynthesis, biotin metabolism, and amino acid metabolism; and plant defense-related pathways, such as phenylpropanoid biosynthesis, flavonoid biosynthesis, stilbenoid, diarylheptanoid and gingerol biosynthesis, phenylalanine metabolism, and the phosphatidylinositol signaling system, which are typical pathways related to plant defense [33]. Phenylpropanoid biosynthesis is the main pathway of phenolic and lignin biosynthesis in plants, and the intermediate products are also substrates for the synthesis of flavonoids, which are secondary metabolites involved in plant defense. Fatty acid related metabolic pathways are closely related to the wound healing of plant tissues [34]. In addition, some DEGs were enriched in the phosphatidylinositol signaling system pathway, which plays a key role in the regulation of cell wall reconstruction during the postharvest morphological development of *Dictyophora indusiata* [33]. We performed a further analysis on the common down-regulated DEGs. The results showed that these down-regulated DEGs were mainly involved in DNA-related pathways, indicating that a series of physiological remodeling took place after the cutting of potato tubers.

Studies have shown that the response of plants to injury at different stages after injury differs. For example, Smith et al. (2004) [32] showed that genes related to aging and death are responsive only at the late stage of poplar wounding. Therefore, we conducted a functional analysis on the specific DEGs at different times after the cutting of potato tubers, and found that there were differences in response to cut-wounding at different times from 0 to 24 h. In this study, the number of specific DEGs at the three time points was the highest at 4 h, the lowest at 12 h, and increased again at 24 h. This result implies a difference in the three stages of potato tubers’ response to cut-wounding. Furthermore, our results showed that the main functions of the specific DEGs at 4 h were enriched in the GO term of ion binding, while the specific DEGs at 12 h were more significantly enriched in nucleic acid-related function terms; the specific DEGs at 24 h were more significantly enriched in the single-organism metabolic process, metabolic process, and catalytic activity terms. These indicated the differences in response to cut-wounding at different stages after cutting in potato tubers. To date, few studies have been conducted on genes specific to different stages after plant tissue damage, and further studies are needed.

### 4.2. Enzymatic Browning-Related Genes

Among the physiological factors limiting the postharvest storage of fresh-cut fruits and vegetables, enzymatic browning plays a major role in reducing the sensory quality and nutritional value of these products [35]. The phenylpropanoid biosynthesis pathway is important in response to biological and abiotic stresses and produces several secondary metabolites, such as phenolic acid, which are substrates of enzymatic browning. Phenolic acid and lignin can cross-link to act as components of the cell wall and are essential for healing the wounded tissues of plants [36]. A study showed that the accumulation of phenolic compounds and increased activities of related enzymes contribute to the early defense against pathogens in plants [37]. In our study, there were 80–101 DEGs enriched in the phenylpropanoid biosynthesis pathway, including genes encoding phenylalanine ammonia-lyase (PAL, EC:4.3.1.24), trans-cinnamate 4-monooxygenase (C4H, EC:1.14.14.91), 4-coumarate--CoA ligase (4CL, EC:6.2.1.12), caffeoylshikimate esterase [EC:3.1.1.-], shikimate O-hydroxycinnamoyltransferase (HQT, EC:2.3.1.133), 5-O-(4-coumaroyl)-D-quinate 3′-monooxygenase (C3′H, EC:1.14.14.96), caffeoyl-CoA O-methyltransferase (CCoAOMT, EC:2.1.1.104), ferulate-5-hydroxylase (F5H, EC:1.14.-.-), cinnamoyl-CoA reductase (CCR, EC:1.2.1.44), coniferyl-aldehyde dehydrogenase (CALDH, EC:1.2.1.68), cinnamyl-alcohol dehydrogenase (CAD, EC:1.1.1.195), β-glucosidase (BG, EC:3.2.1.21), peroxidase (POX, EC:1.11.1.7), and coniferyl-alcohol glucosyltransferase (UGT72E, EC:2.4.1.111). In this pathway, except for a few BG and some POD genes whose expression was down-regulated in response to cut-wounding, all other genes were up-regulated, and there were 15 DEGs with a |log2FC| ≥ 6. Notably, UGT72E was significantly up-regulated only 24 h after cutting. PAL is a rate-limiting enzyme in phenylpropanoid biosynthesis and a key enzyme in phenol synthesis. It catalyzes the first committed step in the phenylpropanoid pathway, producing trans-cinnamic acid, which flows through a proposed biosynthesis route, forming precursors for assembly into suberin polyphenolic(s). Injury induced PAL gene expression and an increase in enzyme activity have been confirmed by numerous studies [38,39]. The overexpression of the PAL gene can increase phenolic biosynthesis in plants, and the inhibition of the expression of the PAL gene decreases phenolic and lignin synthesis [33]. In this study, the expression of all the nine PAL genes was up-regulated in response to cut-wounding, with log2FC values of 1.13–5.24, hinting that the biosynthesis of phenolic compounds in potato tubers was induced by cut-wounding, which was further validated by the result of qRT-PCR (Figure 6). The activity of PAL also has significantly increased at 12 h after cutting (Figure 7).

POD is involved in a variety of physiological processes, including mediating the polymerization of phenolic substances into lignin, thereby enhancing the strength of cell walls [40]. POD activity increases in response to pathogen attack, wounding, and other stresses [40,41]. In this study, the expression of a total of 43 POD genes showed significant changes in response to cut-wounding, and eight had a |log2FC| > 6. POD activity was the highest at 0 h, then decreased, and increased again at 24 h.

PPO is well known to be a key enzyme in plant tissue enzymatic browning. When plant tissues are damaged, they can catalyze the conversion of monophenol or bisphenol to quinones and then combine with intracellular proteins and other macromolecules to produce colored substances, thus leading to browning on the surface of tissues [4]. Enzymatic browning of plant tissues is considered to be a mechanism of plant self-protection because the quinones produced by PPO-catalyzed phenolics have a toxic effect on diseases and insect pests [42]. Furthermore, PPO plays a crucial role in the biosynthesis of secondary metabolites such as aurones and betalins [43]. It also plays an important role in the healing process of plant tissue damage. The transcript level of PPO was consistently up-regulated by mechanical injury, pathogen infection, animal herbivory, and signaling molecules (e.g., JA, SA) [44]. In this study, a total of six unigenes of PPO were detected, which were continuously up-regulated 0–24 h after cutting, which is consistent with our result of qRT-PCR (Figure 6). The activity of PPO also increased in response to cut-wounding (Figure 7).

Oxygen burst is an important feature of plant responses to biotic and abiotic stresses. Under stress conditions, plants modulate their homeostatic mechanism by generating an excess level of ROS, such as H_2_O_2_, hydrogen free radical (OH-), oxygen free radical or superoxide (O2-), in order to survive [45]. CAT maintains cellular H_2_O_2_ homeostasis under environmental stimuli in sweet potatoes [46]. When ROS increase, chain reactions start in which SOD catalyzes the dismutation of superoxide radicals (O2∙−) to molecular oxygen (O_2_) and H_2_O_2_. H_2_O_2_ is then detoxified by CAT, POD, and APX [47]. In this study, four SOD and four CAT genes were detected and showed significant changes in response to cut-wounding. The activity of CAT has higher transcript level and enzyme activity at 0 h after cutting, while it is lower at 4 h. Higher initial CAT activity is conducive to the timely degradation of H_2_O_2_.

### 4.3. Signaling Molecules Biosynthesis Related-Genes Involved in Wound-Response

Plants integrate multiple hormone-response pathways for adaptation to biological and abiotic stress. They can transport the wounding signal to adjacent tissues, inducing changes in related genes. When plants are injured, wounding signals are transmitted to neighboring tissues by phytohormones, including IAA, GA, ET, ABA, JA, SA, and CK [48,49,50]. Studies have shown that ET is involved in various stress responses in plants, including injury, and can induce PAL synthesis [51], which leads to the synthesis of phenolic substances and lignin. The rise of ET is also related to the aging of the organism, which is often directly related to the browning of tissues (e.g., bananas, dates, etc.) [52]. IAA and JA also play an important role in the metabolic response to wounding [53]. SA and JA are key signaling molecules that regulate plant resistance [54]. Mechanical damage provokes the de novo synthesis of PAL in plant tissues, probably by signaling pathways involving compounds such as SA and JA [52]. The systemic wound response also contributes to the rapid synthesis of JA to enhance plant resistance to herbivore and insect attacks [55]. The H_2_O_2_ burst depends on JA production in the plant wounding response. Both wounding and JA treatment led to a significant increase in the activities of plasma membrane NADPH oxidase and PAL [56]. In addition, ABA plays an important role in the healing of potato tuber injury and can stimulate the formation of corking [48,50,57]. Studies have shown that ABA promotes the formation of postharvest potato tuber calli by increasing the activity of PAL and other enzymes and promoting the content of flavonoids and phenols. Furthermore, it can also induce the accumulation of POD gene transcripts in tomato [58]. In addition, the JA pathway positively regulates polyphenol oxidase-based defense against tea geometrid caterpillars in the tea plant (*Camellia sinensis*) [59]. In the present study, we found that numerous up-regulated unigenes were related to the hormone biosynthesis and hormone response pathways, such as the gene encoding 1-aminocyclopropane-1-carboxylate synthase, which is a key gene for ET biosynthesis and is dramatically increased at 4 h after cutting. A gene encoding pyruvate decarboxylase catalyzes thiamine diphosphate to acetaldehyde, which is the substrate of ET biosynthesis. Three genes encoding ent-kaurenoic acid oxidase and gibberellin β-dioxygenase, which were the key genes for GA biosynthesis, increased drastically with a log2FC > 6. Ten unigenes were related to IAA, JA and SA signal transduction. However, for signaling molecules, the sampling time seems to be late in our study, because signaling molecules are produced earlier after cutting, such as 30 min or 1 h. Therefore, it is necessary to further study the earlier response after cutting. Rboh-mediated H_2_O_2_ production, known as an initial response to environmental stresses, is required for the translocation of the systemic signal to distant plant parts, thereby triggering responses of plants to cope with future stress events, including cold, heat, wounding, and salinity [55]. Here, the Rboh genes detected were up-regulated in response to cut-wounding. However, the H_2_O_2_ content decrease gradually, which may be due to the timely degradation of CAT.

### 4.4. Transcription Factor Genes Identified in Response to Cut-Wounding

It is known that, after the application of different abiotic stresses, the signaling molecules induce the production of transcription factors that activate genes coding for enzymes needed for secondary metabolite biosynthesis [60]. These same stresses will encode for enzymes associated with quality attribute changes in a range of biochemical pathways affecting volatile and color production, texture, and flavor [18]. Transcription factors bind to cis-acting elements in the promoters of environmental stress-responsive genes and interact with other transcription factors, subsequently activating or repressing gene expression and playing a central role in the plant response to environmental stresses [61]. In this study, the number of transcription factors found in response to cut-wounding was significantly different at different stages. The results showed that 0–24 h after cutting, the number of differentially expressed transcription factors decreased significantly over time, which indicated that most of the transcription factor genes responded in the early phase after cutting. In this study, the transcription factors detected were divided into 75 types, of which MYB was the most numerous, followed by AP2-EREBP. MYB is one of the most abundant transcription factor families in plants and is involved in plant responses to a variety of biotic and abiotic stresses. Studies have shown that MYB transcription factors are also closely associated with programmed plant death, phenol biosynthesis and enzymatic browning after injury [62]. MYB can regulate the activity of PPO and the biosynthesis of phenols by inducing the expression of PAL genes [63]. R2R3-MYB transcription factors have essential roles in many aspects of plant secondary metabolism, particularly phenylpropanoid metabolism [64]. Studies have shown that MYB transcription factors regulate the expression level of genes by binding to key gene promoters in the phenylpropanoid biosynthesis pathway [65]. In addition, the R2R3 MYB transcription factor has been shown to be a key regulator of flavonoid synthesis in grapes [66]. In our study, the number of MYB transcription factors was the highest, indicating its importance in the response to cut-wounding of potato tubers.

AP2/EREBP are considered transcription factors induced by abiotic stress and are also considered to play a crucial role in the response to wounding [67]. HbEREBP1 expression was down-regulated by mechanical wounding in the laticifers of adult trees, suggesting that HbEREBP1 may be a negative regulator of defense genes in laticifers [67]. In addition, HbEREBP1 may be a key regulator mediating the crosstalk between the ET and JA signaling pathways in the regulation of defense gene expression in laticifers of rubber trees [67,68]. AP2/EREBP transcription factors also play an important role in the JA signaling pathway. Many members of the AP2/EREBP family can be induced by JA signaling molecules to initiate the expression of a series of related genes, thus producing JA-induced physiological effects [69,70]. In this study, in addition to MYB and AP2/EREBP transcription factors, many C3H transcription factors were also found in response to cut-wounding in potato tubers, indicating that C3H transcription factors also play an important role in the potato tuber injury response. Based on the results presented above, most TFs respond to injury at the early stage after the cutting of potato tubers. Therefore, we further selected TFs with a |log2FC| ≥ 6 at 4 h after cutting. The results showed that the selected TFs included three MYB, two AP2-EREBP, two Tify, and one bHLH, LOB, HB, WRKY, E2F-DP, EIL, and MADS gene. The role of these TFs in response to cut-wounding and how to regulate enzymatic browning of potato tubers needs to be studied further.

### 4.5. Hypothetical Model of Potato Tubers in Response to Cut-Wounding

**Figure 8:**
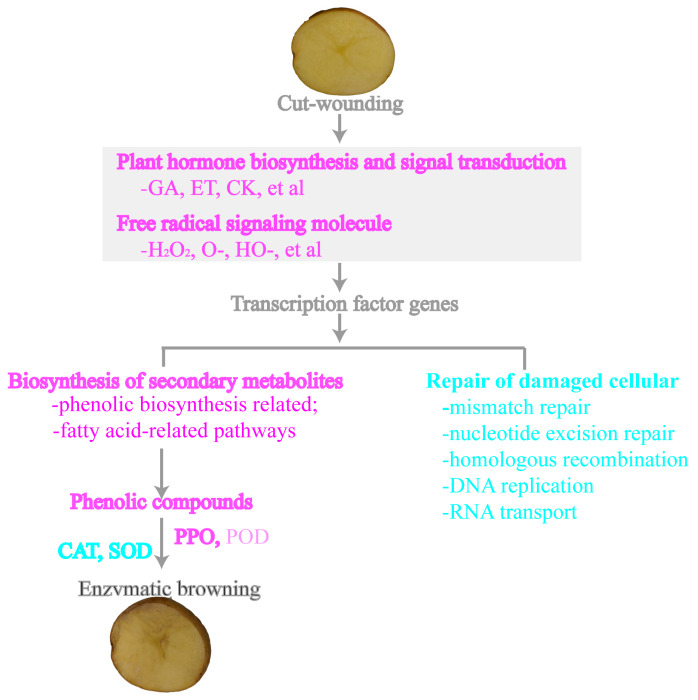
Hypothetical model of potato tubers in response to cut-wounding. Purple and cyan refer to the genes up-regulated and down-regulated in response to cut-wounding.

Based on the above results, we constructed a gene regulatory network hypothetical model of potato tubers in response to cut-wounding (Figure 8). In general, the genes related to secondary metabolism were up-regulated, while the genes related to cellular repair were down-regulated after potato tuber cutting. After potato tubers are cut, the tissues produce chemical signaling molecules, such as ROS and plant hormone (GA, ET, CK, et al.), and induce transcription factors to activate genes coding for enzymes needed for secondary metabolite biosynthesis, further regulating the biosynthesis of secondary metabolites, including phenols and lipids. In addition, the gene’ related enzymatic browning was also activated, such as PPO, POD, and CAT, etc. The higher initial activity of CAT can reduce the enzymatic browning degree caused by PPO and POD through the degradation of H_2_O_2_. Therefore, this study suggests that to improve enzymatic browning, it is not only important to focus on the PPO genes, but also to try to reduce the activity of POD and SOD directly or indirectly, or improve the activity of CAT by gene editing technology popular at present, or by other physical or chemical methods to inhibit the occurrence of enzymatic browning, which is worthy of further attempts. Recent studies have shown that CAT treatment can reduce the enzymatic browning of potato tubers [71].

## 5. Conclusions

In this study, we investigated a series of physiological changes of potato tubers at different times after cutting at the transcriptome level. The results showed that a total of 10,872, 10,449, and 11,880 DEGs were identified at 4 h, 12 h and 24 h after cutting, respectively. More than 87.5% of these DEGs were classified into the categories of BP and MF based on GO analysis. The genes related to the pathways of phenol and fatty biosynthesis, which are responsible for the enzymatic browning and wound healing of potato tubers, were significantly enriched 0–24 h after cutting. Most gene related enzymatic browning, such as PPO, POD, PAL, and Robh of potato tubers up-regulated in response to cutting. Plant hormone biosynthesis and signal transduction related genes, such as GA, CA, ET, IAA, JA, and SA, significantly changed in response to cut-wounding at early stages. In addition, the transcriptional factor genes involved in wound response were the highest in the early stage after cutting. Furthermore, the genes most responsive to injury transcription factors were MYB, followed by AP2-EREBP, C3H and WRKY. To reduce the enzymatic browning of potato tubers, the methods of reducing the activity of POD, SOD and improving the activity of CAT by gene editing technology may be an alternative approach.

This study will provide a new idea with regard to the inhibiting of the enzymatic browning of potato tubers by studying the transcriptome changes of potato tubers after cutting.

## Figures and Tables

**Table 1:** Enrichment of the DEGs with |log2FC| ≧ 6 on KEGG pathways.

Pathways	Gene Number	*p*-Value	Gene ID
Flavonoid biosynthesis	7	0.000261	PGSC0003DMG400003563, PGSC0003DMG400013684, PGSC0003DMG400029621, PGSC0003DMG400029620, PGSC0003DMG400014093, PGSC0003DMG400024643, PGSC0003DMG400019110,
Phenylpropanoid biosynthesis	15	0.00058	PGSC0003DMG400021152, PGSC0003DMG400010465, PGSC0003DMG400014867, PGSC0003DMG400012658, PGSC0003DMG400013684, PGSC0003DMG400030430, PGSC0003DMG400003013, PGSC0003DMG400026575, Novel01675, Novel01676, PGSC0003DMG400001774, PGSC0003DMG400016114, PGSC0003DMG400016113, PGSC0003DMG400015548, PGSC0003DMG400018446
DNA replication	6	0.00465	PGSC0003DMG400024634, PGSC0003DMG403013782, PGSC0003DMG400019109, PGSC0003DMG400008308, PGSC0003DMG400008876, PGSC0003DMG400011837
Zeatin biosynthesis	5	0.005041	PGSC0003DMG400008137, PGSC0003DMG400000277, PGSC0003DMG400005960, PGSC0003DMG402027210, PGSC0003DMG400027200
Taurine and hypotaurine metabolism	3	0.007694	Novel02601, Novel00136, PGSC0003DMG400022764
Butanoate metabolism	4	0.008258	Novel02601, Novel00136, PGSC0003DMG400022764, PGSC0003DMG400025228
Pentose and glucuronate interconversions	8	0.013374	PGSC0003DMG400012640, PGSC0003DMG401019255, PGSC0003DMG402019255, PGSC0003DMG400031791, PGSC0003DMG400031816, PGSC0003DMG400015815, PGSC0003DMG400015230, PGSC0003DMG400029645
Other types of O-glycan biosynthesis	2	0.021717	PGSC0003DMG400021652, PGSC0003DMG400014295
Homologous recombination	5	0.022726	PGSC0003DMG400019109, PGSC0003DMG402010496, PGSC0003DMG400024634, PGSC0003DMG403013782, PGSC0003DMG400008876
Phenylalanine metabolism	9	0.034537	PGSC0003DMG400026575, PGSC0003DMG400010465, PGSC0003DMG400014867, PGSC0003DMG400013684, PGSC0003DMG400030430, Novel01675, Novel01676 PGSC0003DMG400001774, PGSC0003DMG400015548
Mismatch repair	4	0.038656	PGSC0003DMG400019109, PGSC0003DMG400024634, PGSC0003DMG403013782, PGSC0003DMG400008876
Sesquiterpenoid and triterpenoid biosynthesis	3	0.054213	PGSC0003DMG400020939, PGSC0003DMG400017997, PGSC0003DMG400006713
Alanine aspartate and glutamate metabolism	4	0.058281	Novel02601, Novel00136, PGSC0003DMG400022764, PGSC0003DMG400025228
Biosynthesis of unsaturated fatty acids	4	0.058281	PGSC0003DMG400002943, PGSC0003DMG400023833, PGSC0003DMG400023843, PGSC0003DMG400000984
Diterpenoid biosynthesis	3	0.071621	PGSC0003DMG400027645, PGSC0003DMG400001823, PGSC0003DMG400009021
Starch and sucrose metabolism	10	0.081798	PGSC0003DMG400004659, PGSC0003DMG400031816, PGSC0003DMG401019255, PGSC0003DMG402019255, PGSC0003DMG400003013, PGSC0003DMG400029892, PGSC0003DMG400031800, PGSC0003DMG400013809, PGSC0003DMG400031791, PGSC0003DMG400009109
Nitrogen metabolism	3	0.096402	PGSC0003DMG400016996, Novel01004, PGSC0003DMG400008094
Cutin suberine and wax biosynthesis	3	0.107167	PGSC0003DMG402023841, PGSC0003DMG400007405, PGSC0003DMG401023841
Nucleotide excision repair	4	0.114737	PGSC0003DMG400019109, PGSC0003DMG400024634, PGSC0003DMG403013782, PGSC0003DMG400008876
2-Oxocarboxylic acid metabolism	4	0.114737	PGSC0003DMG400006730, PGSC0003DMG400017513, Novel03278, Novel00090
Cyanoamino acid metabolism	3	0.154364	PGSC0003DMG400004660, PGSC0003DMG400003013, Novel00090
Circadian rhythm-plant	3	0.154364	PGSC0003DMG400029621, PGSC0003DMG400019110, PGSC0003DMG400029620
Valine leucine and isoleucine biosynthesis	2	0.184382	PGSC0003DMG400006730, Novel03278
Glucosinolate biosynthesis	1	0.191014	Novel00090
Carotenoid biosynthesis	2	0.271484	PGSC0003DMG400018481, PGSC0003DMG400018480
Monoterpenoid biosynthesis	1	0.272409	PGSC0003DMG400038230
Pyrimidine metabolism	5	0.288408	PGSC0003DMG400019109, PGSC0003DMG400007335, PGSC0003DMG400020112, PGSC0003DMG400024725, PGSC0003DMG400033050
Plant hormone signal transduction	10	0.289881	PGSC0003DMG400028800, PGSC0003DMG400006480, PGSC0003DMG400028254, PGSC0003DMG400019274, PGSC0003DMG400003227, PGSC0003DMG400008950, PGSC0003DMG400005585, PGSC0003DMG400008001, PGSC0003DMG400023678, PGSC0003DMG400003228
β-Alanine metabolism	3	0.298071	Novel02601, Novel00136, PGSC0003DMG400022764
Fatty acid metabolism	4	0.318414	PGSC0003DMG400002943, PGSC0003DMG400023833, PGSC0003DMG400023843, PGSC0003DMG400000984
Degradation of aromatic compounds	1	0.32805	PGSC0003DMG400013684
Glutathione metabolism	4	0.336258	PGSC0003DMG400002171, PGSC0003DMG400002167, PGSC0003DMG400024452 PGSC0003DMG400015726
SNARE interactions in vesicular transport	2	0.367906	PGSC0003DMG400016283, PGSC0003DMG400000733
Vitamin B6 metabolism	1	0.379449	Novel01870
One carbon pool by folate	1	0.395696	PGSC0003DMG400004660
Ubiquitin mediated proteolysis	5	0.464025	Novel02424, PGSC0003DMG400031240 PGSC0003DMG400000540, PGSC0003DMG400029675, PGSC0003DMG401027032
Amino sugar and nucleotide sugar metabolism	4	0.466024	PGSC0003DMG400026853, PGSC0003DMG400031800, PGSC0003DMG400004659, PGSC0003DMG400026854
Stilbenoid diarylheptanoid and gingerol biosynthesis	1	0.498149	PGSC0003DMG400013684
Glyoxylate and dicarboxylate metabolism	2	0.542125	PGSC0003DMG400004660, Novel01867
Histidine metabolism	1	0.583279	PGSC0003DMG400006731
Photosynthesis-antenna proteins	1	0.594203	PGSC0003DMG400007375
Pantothenate and CoA biosynthesis	1	0.594203	PGSC0003DMG400004975
Glycine serine and threonine metabolism	2	0.601955	PGSC0003DMG400004660 Novel01867
Ubiquinone and other terpenoid-quinone biosynthesis	1	0.615203	PGSC0003DMG400013684
Fatty acid elongation	1	0.635119	PGSC0003DMG400000984
Plant-pathogen interaction	5	0.654274	PGSC0003DMG400028800, PGSC0003DMG400026914, PGSC0003DMG400013427, PGSC0003DMG400023125, PGSC0003DMG400006480
Base excision repair	1	0.68891	PGSC0003DMG400019109
Pyruvate metabolism	2	0.709095	PGSC0003DMG400006730, Novel03278
Photosynthesis	2	0.71458	PGSC0003DMG400017556, PGSC0003DMG400017848
Arginine and proline metabolism	2	0.72529	PGSC0003DMG400024452, PGSC0003DMG400017513
Phagosome	2	0.740718	PGSC0003DMG400030627, PGSC0003DMG400000304
Protein export	1	0.755139	Novel00948
Peroxisome	2	0.769364	PGSC0003DMG400007405, PGSC0003DMG403005658
Endocytosis	3	0.789757	PGSC0003DMG400011197, PGSC0003DMG400021197, PGSC0003DMG400013265
Galactose metabolism	1	0.812368	PGSC0003DMG401001316
Glycolysis/Gluconeogenesis	2	0.855078	PGSC0003DMG400006270, PGSC0003DMG401001316
Biosynthesis of amino acids	4	0.881486	PGSC0003DMG400004660, PGSC0003DMG400006730, Novel03278 PGSC0003DMG400017513
Carbon fixation in photosynthetic organisms	1	0.883868	PGSC0003DMG400029406
Purine metabolism	2	0.931195	PGSC0003DMG400019109, PGSC0003DMG400007335
Cysteine and methionine metabolism	1	0.945021	PGSC0003DMG400021426
mRNA surveillance pathway	1	0.965116	PGSC0003DMG400003416
Protein processing in endoplasmic reticulum	2	0.991942	PGSC0003DMG400011197, PGSC0003DMG400016468
Carbon metabolism	2	0.992669	PGSC0003DMG400029406, PGSC0003DMG400004660
Spliceosome	1	0.995473	PGSC0003DMG400011197

## Data Availability

Not applicable.

## Supplementary Materials

The following supporting information can be downloaded at: https://www.mdpi.com/article/10.3390/genes14010181/s1, Figure S1: Correlation matrix between samples; Figure S2: Principal component analysis of samples; Figure S3. Verification of RNA-seq expression via qRT-PCR. Figure S4: GO enrichment of specific DEGs of potato tubers at 4 h (a), 12 h (b) and 24 h (c) after cutting. Table S1: The primers used for real-time PCR in this study; Table S2: The quality of transcriptome data; Table S3: Statistical analysis of assembly reads mapped to the transcript; Table S4: The DEGs of potato tubers at different time after cutting; Table S5: GO and KEGG enrichment of common DEGs at different time after cutting; Table S6: The GO enrichment of specific DEGs at different time after cutting; Table S7: The information of antioxidant enzymes and enzymatic browning-related genes; Table S8: The information of genes with Log2FC more than 6; Table S9: The transcriptional factors in response to cut-wounding at different times.
