# Supplementary figures and images for: A Genome-Wide View of the Transcriptome Dynamics of Fresh-Cut Potato Tubers
Source: Genes (Basel). 2023 Jan 10;14(1):181. doi: 10.3390/genes14010181 (PMC9859442; doi:10.3390/genes14010181)

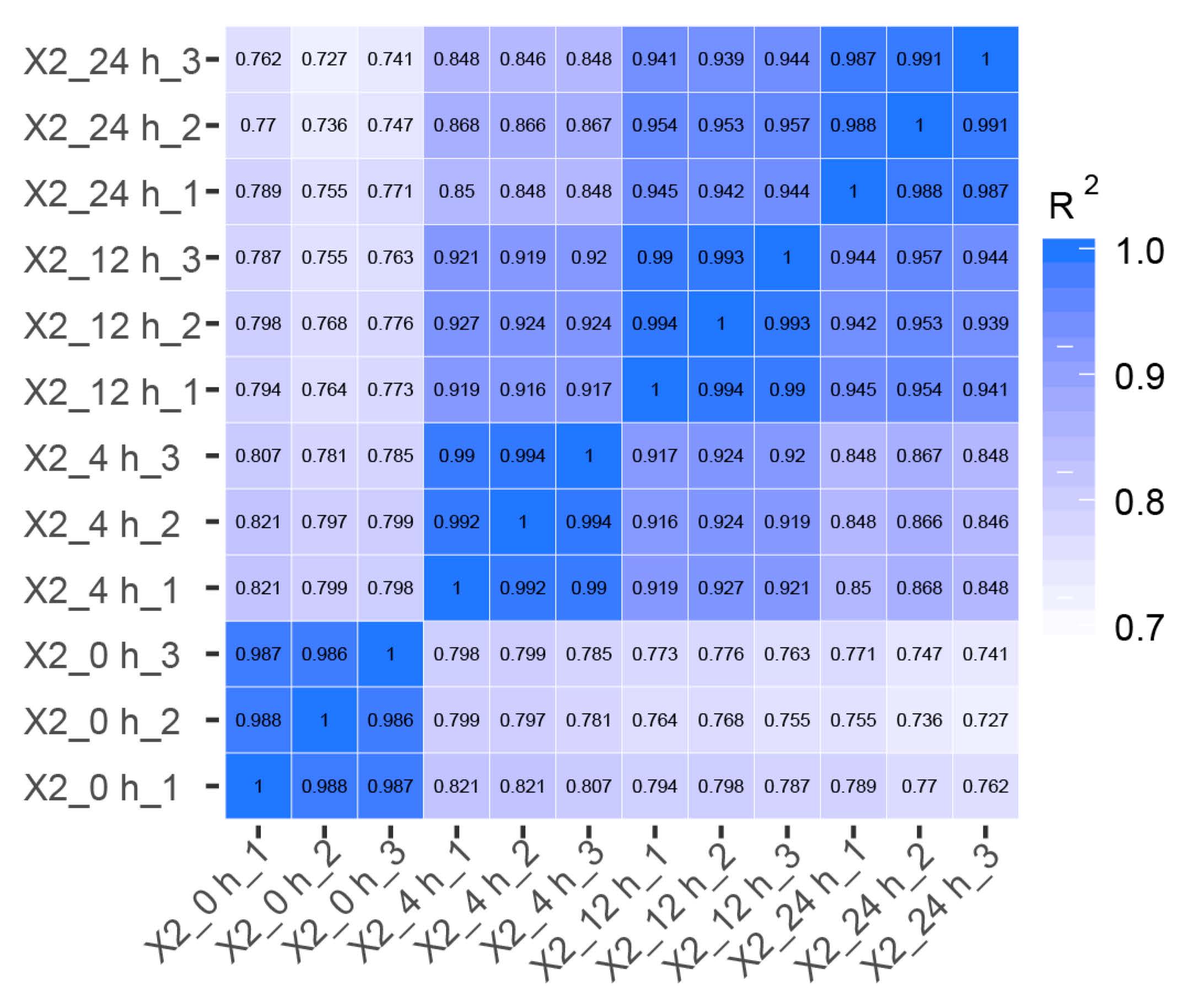

Supplement: Supplementary file 1 [file genes-14-00181-s001.zip › Figure S1 Correlation matrix between samples.jpg]

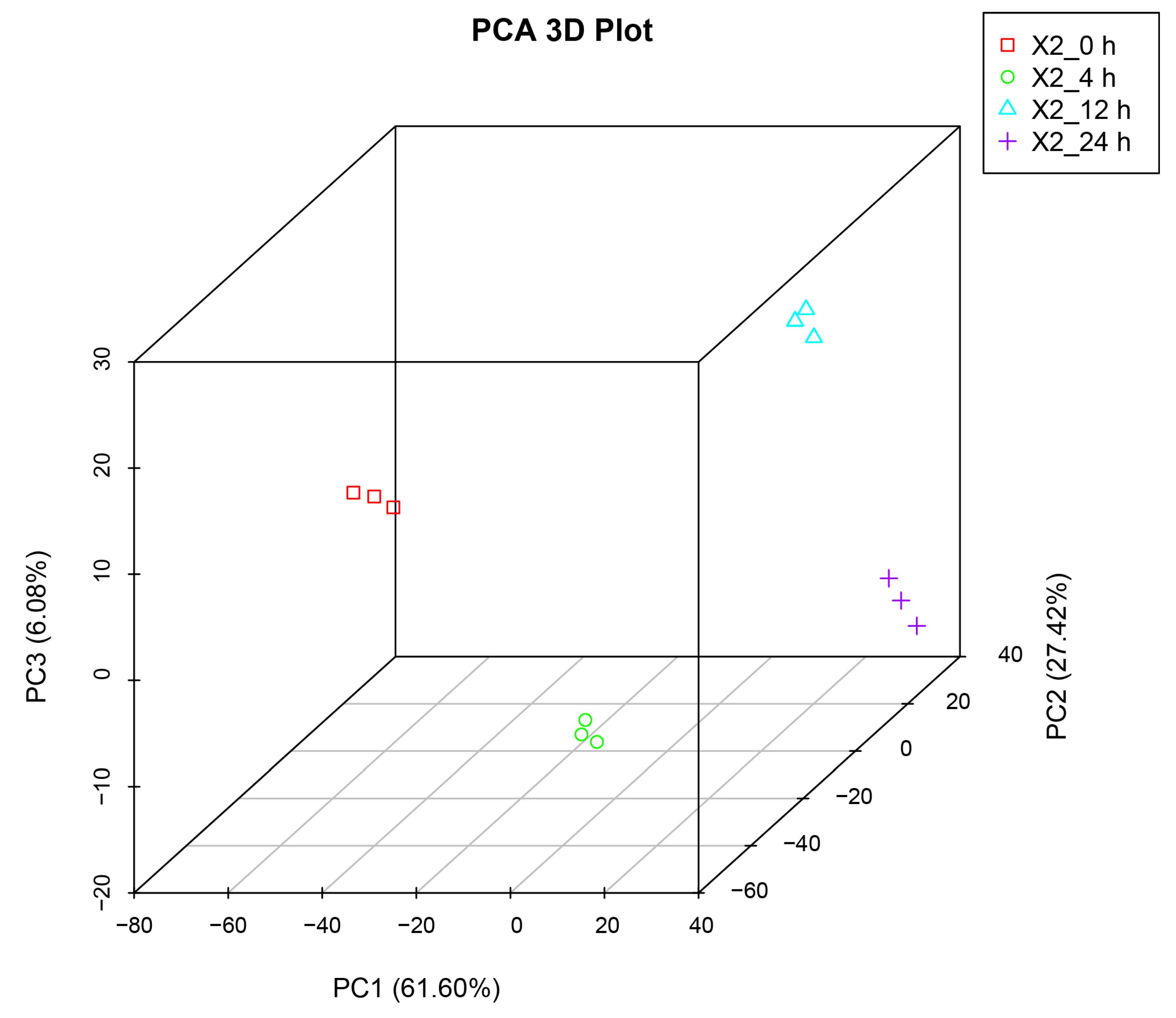

Supplement: Supplementary file 1 [file genes-14-00181-s001.zip › Figure S2 PCA analysis.jpg]

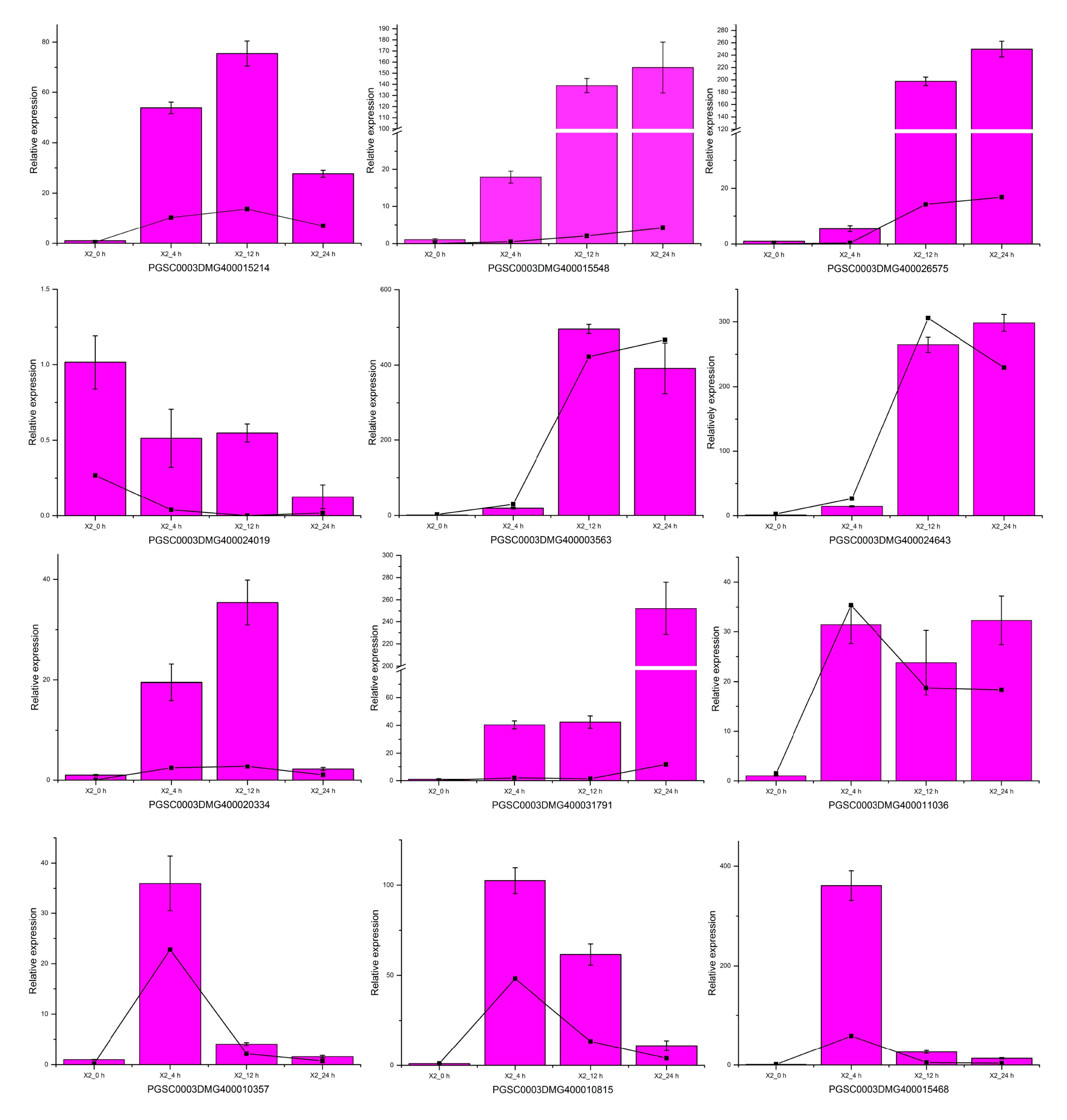

Supplement: Supplementary file 1 [file genes-14-00181-s001.zip › Figure S3 qRT-PCR varification.jpg]

a

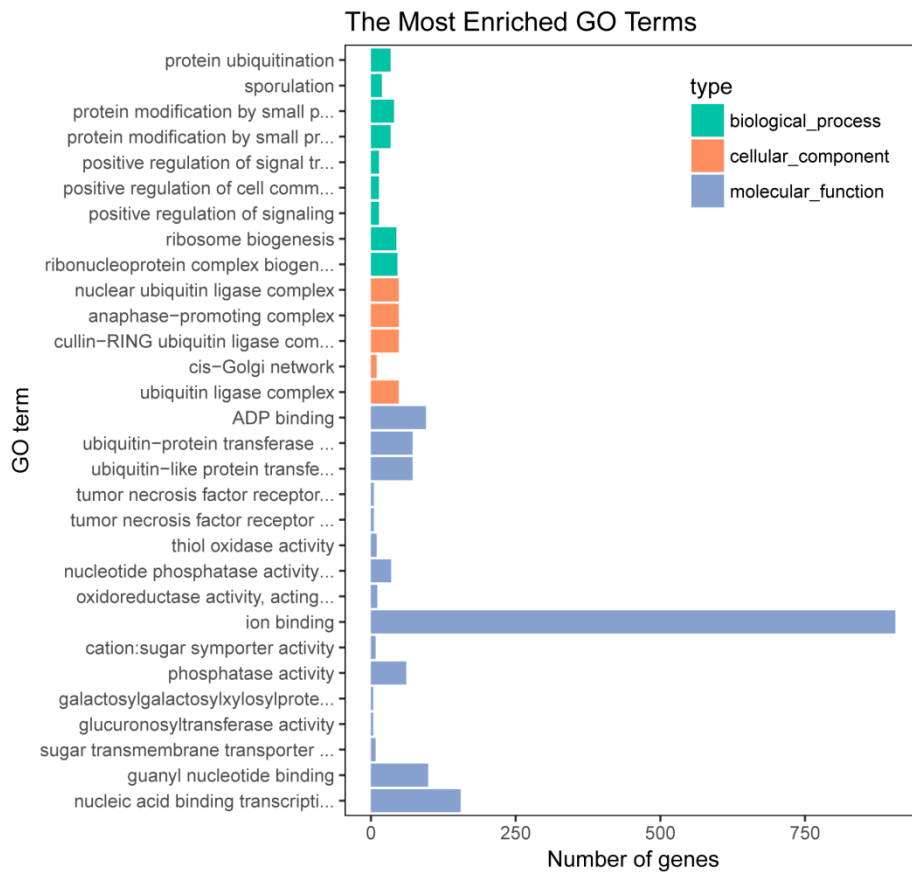

b

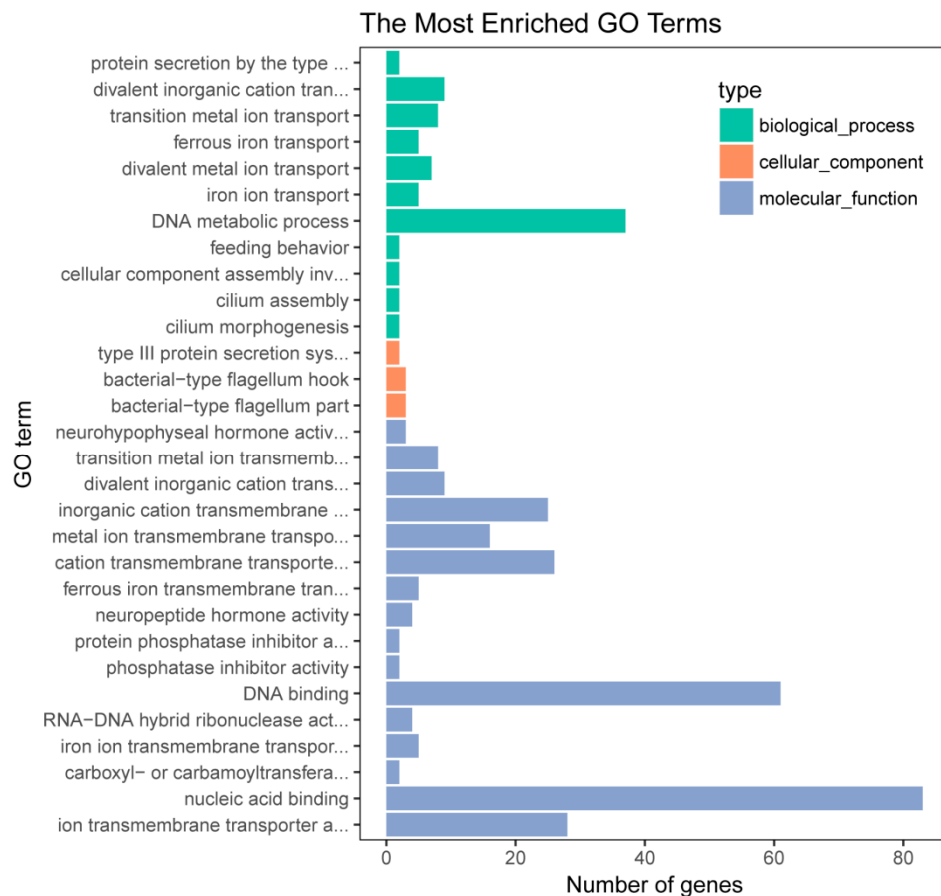

c

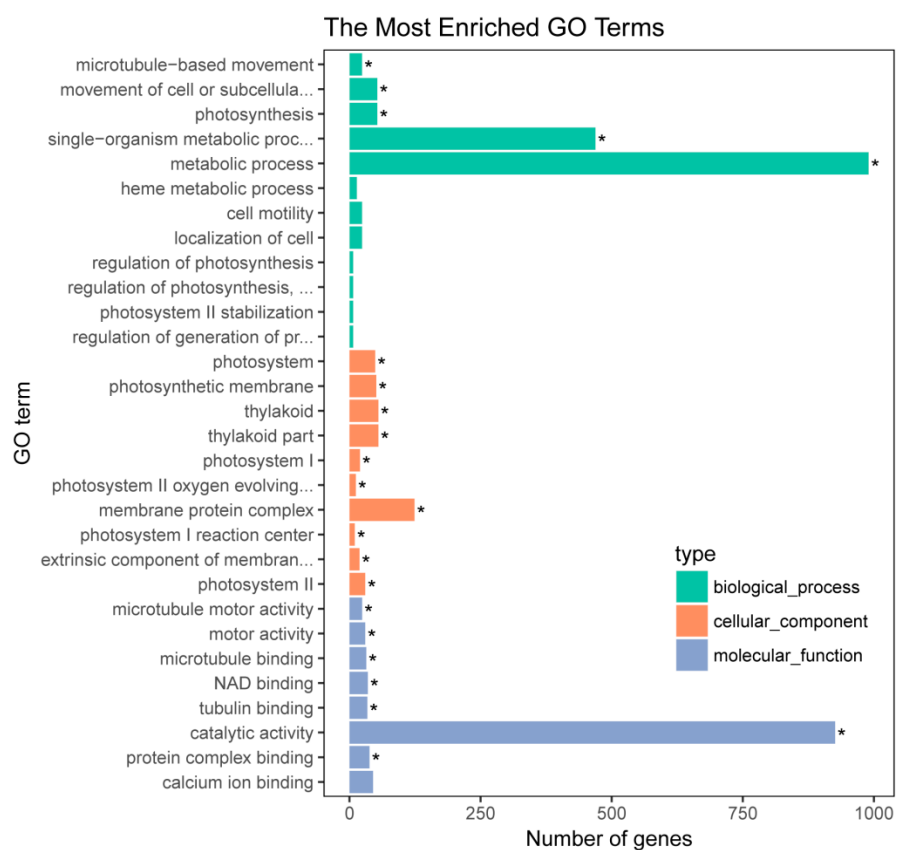

Figure S4 GO enrichment of specific DEGs of potato tubers at 4 h (a), 12 h (b) and 24 h (c) after cut-wounding.

Supplement: Supplementary file 1 [file genes-14-00181-s001.zip › Figure S4 GO enrichment of specific DEGs at different time after cut-wounding.pdf]
